# Supplementary material for: A deep learning model to enhance lung cancer detection using ‘Dual-Branch’ model classification approach
Source: PLoS One. 2026 Jan 9;21(1):e0339404. doi: 10.1371/journal.pone.0339404 (PMC12788669; doi:10.1371/journal.pone.0339404)
Supplement: Attachment — Submitted filename: Response to Reviewers.docx [file pone.0339404.s001.docx]

| **Ref.** | **PONE-D-24-19451** |
| --- | --- |
| **Title** | **A Deep Learning Model to Enhance Lung Cancer Detection using Dual Model Classification Approach** |
| **Journal** | **PLOS ONE** |

**I would like to express my gratitude to the reviewers for their valuable feedback and insightful comments. Their input has greatly enhanced the readability and quality of the work, making it even more impactful.**

| Comments from the reviewers with Authors reply and modifications | | |
| --- | --- | --- |
|  | **COMMENTS** | **Authors Response, Contributions & Reply** |
| **Reviewer #1:** | | |
| 1 | Please write the problem statement or research gap ( What you want to do and why) in introduction clearly. | The Introduction has been revised to highlight the problem statements as below and included within context of Introduction section: -  **Problem Statements:**  Despite significant advancements in machine learning (ML) for lung cancer detection, several challenges limit its clinical adoption. These include limitations in dataset quality and size, difficulties in generalizing models across diverse populations, and the high computational costs associated with ML techniques. Furthermore, the impact of different input modalities, such as images and masks, on classification performance remains underexplored. Therefore, there is a critical need for an optimized ML framework that effectively integrates multiple modalities to enhance classification accuracy and efficiency.  **Research Gap**  The research gaps are  - The limited availability of large dataset.  - High-quality datasets.  - The unique focus on the mask classification component remains unexplored in existing literature. This lack of attention provides an opportunity to investigate and expand upon this novel area.  - Challenges of decision making for selecting and validating which ML models could be the most effective one.  - The high computational costs associated with these technologies.  Addressing these gaps is crucial to improving diagnostic accuracy and patient outcomes. |
| 2 | Based on these gaps, write your contributions very specifically at the end of Introduction in bullet points. | The Introduction has been revised, and the contributions included at the end of the section.  **Contributions**  To overcome these limitations, this research proposes a novel Dual-Model Classification Approach (DMCA) that leverages the complementary strengths of convolutional neural networks (CNNs) and deep neural networks (DNNs). The key contributions of this study include:  • **Systematic Evaluation:** identifying findings from a thorough literature review and assessing the performance of existing machine learning techniques for lung cancer classification. This involves conducting an in-depth evaluation using two different dataset sizes to establish benchmarks and uncover areas for improvement.  • **Dual-Model Framework:** developed and implemented the DMCA, which integrates CNNs for image classification with DNNs for mask classification to enhance overall accuracy and efficiency.  • **Impact Analysis:** Assessed the effects of dataset size and input modality on model performance, with a particular focus on key metrics such as accuracy, sensitivity, and specificity.  • **Advancement in Classification Performance:** Demonstrated that the proposed DMCA achieves superior performance compared to existing approaches, thereby offering a novel methodology that addresses critical gaps in current lung cancer detection and diagnosis strategies.  This study aims to contribute to the field by providing an optimized ML framework that improves lung cancer classification, paving the way for more reliable early detection and better patient outcomes. (Page 5) |
| 3 | You have discussed many papers in Related Works section. please rewrite this section and discuss the papers group wise (make group with similar type of papers and the review those papers analytically). Discuss in such a way so that it justifies your proposal. | The Literature Review section has been rewritten, highlighting the grouping of studies with similar types. The papers are reviewed and discussed with analytical approach to justify the proposed model in this paper. The review has been grouped based on the different models from 2.1 to 2.5, Table 1 provides an overview of previous studies. 1. **Literature Review** 1 **Overview of Machine Learning in Medical Imaging** Lung cancer diagnosis has seen significant advancements through machine learning (ML), with models such as Convolutional Neural Networks (CNN), Support Vector Machines (SVM), and Deep Neural Networks (DNN) demonstrating potential in addressing the limitations of traditional diagnostic methods. 1.1 **CNN-Based Approaches** Convolutional neural networks (CNNs) have revolutionized the field of medical imaging by autonomously learning complex image features. This remarkable capability has made them a cornerstone in lung cancer detection, where innovative approaches continue to evolve. Various studies highlight the diversity in dataset sizes used for training these networks. For example, one investigation by Al-Yasriy et al. (2020) relied on a modest collection of 110 images, whereas da Silva et al. (2016) employed a substantially larger dataset comprising 8,296 images. Tekade and Rajeswari (2018) also incorporated multiple datasets including the renowned LIDC-IDRI and LUNA16 but did not specify the overall number of images used. CT scans have emerged as the primary input modality in these research efforts, with advanced segmentation techniques like U-Net and maximum intensity projection (MIP) significantly enhancing feature extraction. These methodological refinements have translated into varying performance metrics: Al-Yasriy et al. (2020) achieved an accuracy of 93.55% with a 70/30 data split, while Tekade and Rajeswari (2018) reported a 95% accuracy using a 3D VGG-like model. In contrast, da Silva et al. (2016) recorded an accuracy of 82.3% using LIDC-IDRI data. Interestingly, the findings suggest that as the sample size increases, there may be a tendency toward reduced accuracy, hinting at the possibility of classification bias when larger, more heterogeneous datasets are involved. 1.2 **SVM-Based Approaches** Support Vector Machines (SVMs), which are primarily utilized for binary classification tasks, have proven to be effective in distinguishing between benign and malignant lung nodules (Al-Ragab et al., 2023). These methods have been applied across a range of studies, with datasets varying considerably in size. For instance, Rendon-Gonzalez and Ponomarev (2016) worked with a relatively small sample of just 70 images, while Nascimento et al. (2012) employed a moderate dataset consisting of 73 images and achieved an accuracy of 92.78%. Other studies, such as those conducted by Makaju et al. (2018b), processed grayscale images from larger databases, such as LIDC-IDRI, which were compressed to JPEG format for texture analysis and achieved an accuracy of 92% based on 16 training images and 5 validation nodules. In terms of performance, the results across these studies reflect the effectiveness of SVMs in handling lung nodule classification. Nascimento et al. (2012) reported an accuracy of 92.78%, while Makaju et al. (2018b) achieved an accuracy of 92% based on 16 training images and 5 validation nodules. Krewer et al. (2013) also demonstrated the usefulness of SVMs, achieving an accuracy rate of around 90.01% from 33 sample images used in their study. Despite the promising outcomes, it is crucial to note the limited size of many datasets involved in these investigations and the various preprocessing techniques employed. These factors underscore the growing need for standardized practices in dataset management and pre-processing to improve the consistency and scalability of these approaches in clinical applications. for 9 seconds Support Vector Machines (SVMs) have emerged as a pivotal tool in the binary classification of lung nodules, effectively distinguishing between benign and malignant cases. In the literature, various studies have employed relatively small datasets to validate the performance of these models. These modest sample sizes underscore the challenges of working with limited data in medical imaging research. Researchers have predominantly utilized texture descriptors and grayscale image conversion to prepare the input data for SVM classification. A notable example is the work of Makaju et al. (2018b), who processed JPEG grayscale images extracted from the LIDC-IDRI dataset. This approach aimed to distill essential features from the images, thereby enhancing the SVM's ability to make accurate distinctions between benign and malignant nodules. Despite these encouraging results, the overall findings indicate that the small and variable dataset sizes, along with differences in preprocessing techniques, highlight a pressing need for standardized practices. Such standardization is essential to enhance the reproducibility and reliability of SVM-based diagnostic methods in lung nodule classification. 1.3 **DNN-Based Approaches** Deep neural networks (DNNs), known for their complex structures involving multiple nonlinear processing layers, have shown significant potential in the diagnosis of lung cancer. These networks have been applied across studies with varying sample sizes, reflecting the diversity in research approaches. For instance, Kuruvilla and Ganapathi (2013) utilized a modest dataset of 110 images, while da Silva et al. (2016) employed a much larger dataset containing 8,296 images. The study by Lakshmanaprabu et al. (2019) also investigated DNNs, working with a dataset of just 100 images. These varying sample sizes illustrate a broader trend in lung cancer detection research, where dataset size significantly influences model training and evaluation. In terms of input modality, DNNs predominantly work with CT images, serving as the foundation for processing in these studies. Some studies, notably by Lakshmanaprabu et al. (2019), have delved deeper into optimizing the architecture of the networks to improve performance. Their work on developing an "Optimal DNN" reflects ongoing efforts to enhance the efficacy of DNNs in medical imaging. The performance outcomes reported across these studies reveal differences tied to dataset size. Kuruvilla and Ganapathi (2013) achieved a commendable accuracy of 90.3% with their 110-image dataset, while da Silva et al. (2016) attained an accuracy of 82.3% with the larger LIDC-IDRI dataset. In comparison, Lakshmanaprabu et al. (2019) obtained a high accuracy of 94.56% using just 100 images. Such variations in sample sizes offer insight into the scalability of DNN-based approaches and the challenges posed by larger, more heterogeneous datasets. A notable trend that emerges from these findings is that smaller datasets tend to yield higher accuracy, whereas larger datasets introduce greater variability, which may be indicative of challenges in generalizing across more extensive and varied training data. In these studies, computed tomography (CT) images have served as the primary input modality, providing detailed anatomical information crucial for accurate diagnosis. Some investigations have ventured beyond standard architectures by exploring optimized network designs. A notable example is the work of Lakshmanaprabu et al. (2019), who introduced an Optimal DNN architecture specifically tailored for lung cancer diagnosis, aiming to enhance feature extraction and classification performance. 1.4 **Other Models** Recent developments in lung cancer detection have introduced several innovative approaches, particularly focusing on hybrid and specialized techniques. One remarkable category involves VGG-based models, which have demonstrated impressive results, particularly on small datasets. For example, Sheriff et al. (2021) achieved a perfect accuracy of 100% using a modest dataset of just 18 augmented images. On the other hand, Geng et al. (2019) leveraged a larger dataset of 1,937 images, incorporating dilated convolution, to achieve a noteworthy accuracy of 99.23%. These studies illustrate the potential of VGG-based architectures, though the small sample sizes, particularly in Sheriff et al.'s work, highlight the need for further validation on more extensive datasets. Another promising architecture in lung cancer detection is ResNet, a deep residual network known for its ability to build more complex models while mitigating the vanishing gradient problem. Nakrani et al. (2020) applied ResNet to the LIDC-IDRI dataset, obtaining an accuracy of 95.24% using 500 CT scans with a 70/30 data split. This suggests that deep architectures like ResNet can perform well with relatively moderate dataset sizes, contributing to improved diagnostic outcomes. In addition to these conventional models, other specialized techniques have also garnered attention. Subash and Kalaivani (2024) proposed a dual-stage approach combining U-Net and custom CNN architectures. This hybrid model achieved 93.3% accuracy on a mixed dataset of 1,010 images, showcasing the power of synergizing architectures to enhance performance. Meanwhile, Maleki et al. (2021) introduced a K-Nearest Neighbors (KNN) classifier combined with genetic algorithms, which led to a strong accuracy of 96.2%. This further highlights the versatility of machine learning techniques in solving complex diagnostic challenges in medical imaging. Despite the promising results from these advanced and hybrid techniques, the key observation remains clear: while architectures like VGGNet and ResNet show great promise, they still require validation on larger datasets to confirm their generalizability and reliability in real-world clinical settings. Recent advancements in lung cancer detection have seen the emergence of several innovative approaches that combine hybrid and specialized techniques. Among these, models based on the VGG architecture have demonstrated exceptional performance. In parallel, ResNet-based methodologies have also shown promise. Nakrani et al. (2020) applied a ResNet approach to the LIDC-IDRI dataset, utilizing 500 CT scans with a 70/30 split between training and testing, and achieved an accuracy of 95.24%. This finding underscores the potential of deeper network architectures in managing the complex feature extraction necessary for accurate lung cancer diagnosis. Collectively, these studies highlight that while advanced architectures such as VGGNet and ResNet offer significant promise for lung cancer detection, further validation on larger and more diverse datasets is essential. This continued exploration and validation will be critical to ensure that these sophisticated techniques can reliably support clinical decision-making in real-world settings. **Summary** **1- The latest computational models in lung cancer** The below latest studies highlight a multifaceted approach to lung cancer diagnosis using deep learning, with an emphasis on integrating imaging data, IoMT (Internet of Medical Things) inputs, and advanced optimization techniques and can be broadly summarized as follows: - Faruqui et al. (2023) introduce Healthcare-As-A-Service (HAAS) via HAASNet, a cloud-compatible CNN that, when combined with IoMT data, achieves an accuracy of 96.07% and robust performance metrics (precision: 96.47%, recall: 95.39%, F1-score: 94.81%). Despite these promising results, the transition from research to real-world application remains a challenge, particularly in terms of usability. - Faruqui et al. (2021) earlier proposed LungNet, a 22-layer CNN that merges CT image features with IoT data. This model classifies lung cancer into five classes with 96.81% accuracy and a low false positive rate (3.35%), while also distinguishing early-stage sub-classes with 91.6% accuracy. However, issues such as potential misclassification in early-stage cancers and variability across different populations were noted. - Mohamed et al. (2023) developed EOSA-CNN, a hybrid model where the Ebola Optimization Search Algorithm is used to fine-tune CNN weights and biases. The model reached a classification accuracy of 93.21% on the IQ-OTH/NCCD lung cancer dataset, though its performance is limited by data scarcity, imbalanced classes, and increased computational complexity. - Bushra et al., (2023c) applied a Capsule Network-based approach (LCD-Capsule Network) using 4,335 CT images from the Lung Image Database Consortium (LIDC) to detect abnormal lung patches. While this study did not specify limitations, similar research typically faces challenges such as dataset bias and the need for substantial computational resources. - Wang (2022) reinforces the notion that CNN-based methods are superior for tasks involving segmentation, detection, and classification in early lung cancer detection, consolidating the role of deep learning in this domain. - Asuntha & Srinivasan (2020) proposed the FPSOCNN, which integrates Fuzzy Particle Swarm Optimization with CNNs to reduce computational complexity while effectively classifying lung nodules. Although specific numerical performance metrics were not detailed, the model shows significant promise; common issues like overfitting and generalizability remain to be addressed. - Khondoker & Yousuf (2023) explore the use of sequence and relation classification models to extract argumentative components from clinical trial abstracts on lung cancer immunotherapy. While sequence classification achieved satisfactory results, the relation classification model requires more extensive datasets and further refinement, particularly in identifying nuanced relationships. **Overall Synthesis:** These studies collectively demonstrate significant progress in lung cancer diagnosis using deep learning techniques, particularly CNN-based architectures enhanced by IoMT integration and metaheuristic optimization. They report high classification accuracies (typically exceeding 93%), yet share common limitations such as potential misclassification in early stages, dataset bias, computational demands, and challenges in adapting research findings to real-world settings. This trajectory suggests that while deep learning offers powerful tools for lung cancer diagnosis, further research is essential to improve model robustness, scalability, and clinical applicability. **Table 1** summaries the latest studies in lung cancer. Table 1 - The latest computational models in lung cancer **Reference** \| **ML Model** \| **Accuracy Results** \| **Sample Size** \| **Dataset Name**; Faruqui et al., (2023) \| HAASNet (CNN) \| 96.07% \| Not specified \| Not specified; Faruqui et al., (2021) \| LungNet (22-layer CNN) \| 96.81% \| 525,000 images \| Not specified; Mohamed et al., (2023) \| EOSA-CNN \| 93.21% \| Not specified \| IQ-OTH/NCCD; Bushra et al., (2023) \| LCD-Capsule Network \| Not specified \| 4,335 CT images \| LIDC; Wang (2022) \| CNN-based methods \| Not specified \| Not specified \| Not specified; Asuntha & Srinivasan (2020) \| FPSOCNN \| Not specified \| Not specified \| Not specified; Khondoker & Yousuf (2023) \| Sequence classification model. Relation classification model \| Not specified \| Not specified \| abstracts from PubMed **2- Datasets used in computational modes** While ML models have demonstrated impressive performance in lung cancer detection, there is a lack of standardization in dataset sizes and preprocessing techniques, **Table 2** provides an overview of previous studies, emphasizing their accuracy outcomes and the quantity of sample images analyzed. Table 2 - Comparison of Sample Sizes in Various Studies **Reference** \| **ML Model** \| **Accuracy Results** \| **Dataset Sample Size**; **CNN** \| \| \|; Al-Yasriy et al. (2020) \| CNN \| 93.55% \| 110 images; da Silva et al. (2016) \| CNN \| 82.30% \| 8,296 images; Tekade & Rajeswari (2018) \| CNN (3D VGG-like) \| 95% \| Not specified (LIDC-IDRI & LUNA16 datasets); **SVM** \| \| \|; Nascimento et al. (2012) \| SVM \| 92.78% \| 73 images; Makaju et al. (2018b) \| SVM \| 92% \| 16 training images, 5 validation nodules (LIDC-IDRI dataset); Krewer et al. (2013) \| SVM \| 90.01% \| 33 images; Rendon-Gonzalez & Ponomarev (2016) \| SVM \| Not reported \| 70 images; **DNN** \| \| \|; Kuruvilla & Ganapathi (2013) \| DNN \| 90.30% \| 110 images; da Silva et al. (2016) \| DNN \| 82.30% \| 8,296 images; Lakshmanaprabu et al. (2019) \| DNN (Optimal DNN) \| 94.56% \| 100 images; **Other Models** \| \| \|; Sheriff et al. (2021) \| VGG \| 100% \| 18 augmented images; Geng et al. (2019) \| VGG (dilated convolution) \| 99.23% \| 1,937 images; Nakrani et al. (2020) \| ResNet \| 95.24% \| 500 CT scans (LIDC-IDRI dataset); Subash & Kalaivani (2024) \| Hybrid (U-Net + CNN) \| 93.30% \| 1,010 images; Maleki et al. (2021) \| KNN + Genetic Algorithm \| 96.20% \| Not specified In order to address these identified gaps, we aim to develop a novel Dual-modal approach The Dual-Modal Classification Approach (DMCA). Our objective with DMCA is to navigate the issues related to the absence of mask image types and the necessity for large sample sizes, all while maintaining a high degree of diagnostic accuracy with consistency. DMCA hold potential but demand significant computational resources and optimization and should emphasize: 1. Standardized datasets with larger, diverse samples. 2. Exploration of dual-modal techniques to enhance diagnostic accuracy. 3. Benchmarking models on consistent metrics to improve comparability. |
| 4 | No need to write the very generic contents of CNN, SVM and DNN in section 2. | Section 2 has been re-written and merged with the following newly revised contents.  The Literature Review has been revised as indicated in Point #3. |
| 5 | How did you do the pre processing? Have you done any augmentation? | **The Pre-Processing is conducted as: -** the CT image preprocessing has been applied, during preprocessing, a Gabor filter was applied to the images to highlight edges, suppress noise, and detect fine structural details. These preprocessing techniques ensured that the images, maintained at their original resolution of 512 × 512 pixels, were standardized and clarified.  **Data augmentation** techniques are typically applied when the original dataset is insufficient, the study lacks a controlled environment, computational constraints arise, there is a potential risk of overfitting, the data's precision is weak, or the robustness of the models is uncertain.  However, in this research, data augmentation techniques were deliberately not utilized, ensuring that the experiments were conducted both efficiently and effectively |
| 6 | Why did you concatenate output of CNN with DNN ? Please justify clearly. | The CNN component provides robust spatial feature extraction from the entire CT image, while the DNN component capitalizes on the detailed and focused information provided by the masks. Evidence from investigations show that the enhanced performance of the DMCA model is attributable to its dual-modal architecture that effectively integrates the strengths of both CNN and DNN with a significant improvement of performance (For Experiment 1, DMCA achieved an average accuracy of 91.21% with an F1 score of 91.18%, recall of 91.83%, and precision of 90.53%), whereas in Experiment 2, the DMCA model improved to an average accuracy of 98.04% with an F1 score of 98.01%, recall of 98.66%, and precision of 97.37%). The concatenation of these complementary features prior to classification with the SoftMax layer results in a comprehensive representation that drives the high accuracy observed.  The concatenation of these complementary features creates a shared feature space that allows the final SoftMax classifier to achieve better discrimination between benign and malignant nodules.  The fusion created from the concatenation stage generate a unified feature vector that combined both the spatial information captured by the CNN and the detailed nodule features extracted by the DNN. The resulting vector was then passed to a SoftMax classifier, which produced probabilistic outputs corresponding to six cancer severity classes ranging from non-cancerous (0) to various degrees of malignancy (1–5), (Page 26, 2nd Paragraph).  “After processing through their respective networks, the outputs from the CNN and DNN branches were merged using a concatenation layer. This fusion created a unified feature vector that combined both the spatial information captured by the CNN and the detailed nodule features extracted by the DNN. The resulting vector was then passed to a SoftMax classifier, which produced probabilistic outputs corresponding to six cancer severity classes ranging from non-cancerous (0) to various degrees of malignancy (1–5) “. |
| 7 | What is the weakness of your model ? Please mention it in discussion and conclusion sections. | **The Limitations are: -**  **"In Abstract "**  There are certain limitations were observed, such as the high computational requirements stemming from large sample sizes, the constrained information provided by segmentation masks, and the presence of potential biases in the dataset. These challenges hinder the model's ability to generalize effectively. Future research should aim to enhance image quality, broaden the scope of datasets, and overcome segmentation-related constraints to make further progress in lung cancer detection.  **"In Discussion"**  Key limitations include high computational demands, limited segmentation mask information, and dataset biases, which hinder model generalization. Future efforts should focus on enhancing image quality, expanding datasets, and addressing segmentation challenges to advance lung cancer detection. (Page 33, Last Paragraph).  **"In Conclusion and Future Work"**  limitations were encountered, including computational demands due to large sample sizes, limited information from segmentation masks, and potential biases in the dataset. These challenges restrict the model's generalization. Future work should focus on improving image quality, expanding datasets, and addressing segmentation limitations to further advance lung cancer detection. (Page 36, 2nd Paragraph) |
| 8 | Show your results of Epochs vs Validation Accuracy. | The results of Epochs vs validation accuracy are shown in figures below  our evaluation shows that the DMCA model significantly outperforms single-model approaches across multiple performance metrics, regardless of the sample size. The dual-modal approach, which combines CNN and DNN outputs, provides a robust framework for lung cancer classification. Compared to previous studies that used much smaller sample sizes, our study demonstrates that larger, more diverse datasets can be effectively leveraged to improve model accuracy. Moreover, the integration of different input modalities not only enhances overall performance but also mitigates the limitations inherent in single-modal models. These findings underscore the potential of dual-modal strategies for developing advanced, reliable computer-aided diagnostic systems for lung cancer.  Please refer to "Figure 4 - Comparison of Metrics Results for all Models for Experiment 1" and "Figure 5 - Comparison of Metrics Results for all Models for Experiment 2" in Pages, 34 and 35 |
| 9 | Deep learning for lung Cancer detection and classification (2020), b) Healthcare As a Service (HAAS): CNN-based cloud computing model for ubiquitous access to lung cancer diagnosis (2023), c) Automatic detection and classification of lung cancer CT scans based on deep learning and ebola optimization search algorithm (2023), d) LungNet: A Hybrid Deep-CNN Model for Lung Cancer Diagnosis Using CT and Wearable Sensor-based Medical IoT Data (2021), e) LCD-capsule network for the detection and classification of lung cancer on computed tomography images (2023), f) Argument Mining on Clinical Trial Abstracts on Lung Cancer Patients (2023) | These studies have been included in the Paper. **Reference** \| **ML Model** \| **Accuracy** \| **Sample Size** \| **Dataset Name**; Faruqui et al. (2023) \| HAASNet (CNN) \| 96.07% \| Not specified \| Not specified; Faruqui et al. (2021) \| LungNet (22-layer CNN) \| 96.81% \| 525,000 images \| Not specified; Mohamed et al. (2023) \| EOSA-CNN \| 93.21% \| Not specified \| IQ-OTH/NCCD; AR et al. (2023) \| LCD-Capsule Network \| Not specified \| 4,335 CT images \| LIDC; Wang (2022) \| CNN-based methods \| Not specified \| Not specified \| Not specified; Asuntha & Srinivasan (2020) \| FPSOCNN \| Not specified \| Not specified \| Not specified; Khondoker & Yousuf (2023) \| Sequence and relation classification models \| Not specified \| Not specified \| Not specified The above table, and details below listed latest studies highlight a multifaceted approach to lung cancer diagnosis using deep learning, with an emphasis on integrating imaging data, IoMT (Internet of Medical Things) inputs, and advanced optimization techniques and can be broadly summarized as follows: • Faruqui et al. (2023) introduce Healthcare-As-A-Service (HAAS) via HAASNet, a cloud-compatible CNN that, when combined with IoMT data, achieves an accuracy of 96.07% and robust performance metrics (precision: 96.47%, recall: 95.39%, F1-score: 94.81%). Despite these promising results, the transition from research to real-world application remains a challenge, particularly in terms of usability. • Faruqui et al. (2021) earlier proposed LungNet, a 22-layer CNN that merges CT image features with IoT data. This model classifies lung cancer into five classes with 96.81% accuracy and a low false positive rate (3.35%), while also distinguishing early-stage sub-classes with 91.6% accuracy. However, issues such as potential misclassification in early-stage cancers and variability across different populations were noted. • Mohamed et al. (2023) developed EOSA-CNN, a hybrid model where the Ebola Optimization Search Algorithm is used to fine-tune CNN weights and biases. The model reached a classification accuracy of 93.21% on the IQ-OTH/NCCD lung cancer dataset, though its performance is limited by data scarcity, imbalanced classes, and increased computational complexity. • AR et al. (2023) applied a Capsule Network-based approach (LCD-Capsule Network) using 4,335 CT images from the Lung Image Database Consortium (LIDC) to detect abnormal lung patches. While this study did not specify limitations, similar research typically faces challenges such as dataset bias and the need for substantial computational resources. • Wang (2022) reinforces the notion that CNN-based methods are superior for tasks involving segmentation, detection, and classification in early lung cancer detection, consolidating the role of deep learning in this domain. • Asuntha & Srinivasan (2020) proposed the FPSOCNN, which integrates Fuzzy Particle Swarm Optimization with CNNs to reduce computational complexity while effectively classifying lung nodules. Although specific numerical performance metrics were not detailed, the model shows significant promise; common issues like overfitting and generalizability remain to be addressed. • Khondoker & Yousuf (2023) explore the use of sequence and relation classification models to extract argumentative components from clinical trial abstracts on lung cancer immunotherapy. While sequence classification achieved satisfactory results, the relation classification model requires more extensive datasets and further refinement, particularly in identifying nuanced relationships. |
| **Reviewer #2:** | | |
| 1 | Overall, the manuscript contains grammatical flaws. The manuscript need an extensive proofread to address the flaws. | The native English speaker has checked and proofread the submitted this version. |
| 2 | Abstract need to be presented in the format: Background, Objectives, Methodology, Findings, and Implications. | The abstract has been revised to address the Background, Objectives, Methodology, Findings, and Implications  **ABSTRACT**  **Background:** Lung cancer remains one of the deadliest forms of cancer, affecting millions globally. Early detection and accurate classification are crucial for improving survival rates. Computed Tomography (CT) plays a significant role in lung cancer diagnosis. However, previous studies have struggled with challenges such as limited sample availability and constraints in input modalities, negatively impacting model performance.  **Objectives:**  This study aims to address the limitations of existing lung cancer classification models by introducing the **Dual Model Classification Approach (DMCA)**. The key objectives are:   - To enhance lung cancer detection accuracy by integrating both image and mask data. - To assess the impact of sample size and dual-input modalities on classification performance.   **Methodology**   - A **two-stage classification strategy (DMCA)** was developed, combining image and mask data to improve detection capabilities. - Two comparative experiments were conducted using the **LIDC-IDRI dataset** with different data sizes to evaluate the model's effectiveness. - The model's performance was measured using **accuracy and F1-score** across different sample sizes.   **Findings**   - The DMCA demonstrated **91.21% accuracy and 91.18% F1-score** with the smaller dataset. - With the larger dataset, the model achieved **98.04% accuracy and 98.01% F1-score**, indicating improved detection capability and scalability. - Despite these promising results, the study identified certain limitations, including:   - **High computational requirements** due to large sample sizes.   - **Limited information in segmentation masks**, which can restrict model performance.   - **Potential biases in the dataset**, affecting generalizability.   **Implications**   - The DMCA represents a **significant advancement in lung cancer diagnosis**, demonstrating the potential for improved detection and classification. - The findings emphasize the need for **higher-quality imaging, broader datasets, and improved segmentation techniques** to enhance model generalizability. - The approach could contribute to **more effective, scalable, and lifesaving interventions** in lung cancer care.   **Abstract**  Cancer remains a life-threatening global challenge, with lung cancer ranking among the most devastating forms, impacting millions annually. Early detection and accurate classification are essential for improving patient survival rates, and computed tomography (CT) has become a critical tool in lung cancer diagnosis.  Despite advancements, previous studies have faced notable challenges, particularly a shortage of available samples and limitations in input modalities, both of which hinder model performance. Addressing these issues, the objective of this research is to take above mentioned challenges through develop a new mode: the Dual Model Classification Approach (DMCA), a two-stage strategy that integrates image and mask data to enhance detection accuracy and scalability to achieve a better accuracy and performance. We conducted two comparative experiments using the LIDC-IDRI dataset with varying data sizes to evaluate the impact of sample size and dual-input modalities. It is found that the DMCA achieved remarkable results, including 91.21% accuracy and 91.18% F1-score in the smaller dataset and 98.04% accuracy and 98.01% F1-score in the larger dataset. This demonstrates the model's improved discriminative power and potential for detecting subtle lung cancer patterns.  However, limitations were encountered, including computational demands due to large sample sizes, limited information from segmentation masks, and potential biases in the dataset. These challenges restrict the model's generalization. Future work should focus on improving image quality, expanding datasets, and addressing segmentation limitations to further advance lung cancer detection.  The DMCA represents a significant step forward in improving the performance and scalability of diagnostic tools, offering the potential for more effective and lifesaving interventions in lung cancer care. |
| 3 | The introduction part should include motivation and contributions of the study. | The Introduction has been revised to clearly highlight the Motivation and Contribution:-  **Motivation**  The integration of machine learning (ML) in medical imaging presents a promising avenue for improving the early detection and classification of lung cancer. However, several challenges hinder the clinical adoption of ML-based techniques. These challenges include:  • The limited availability of large, high-quality datasets.  • Difficulties in selecting and validating the most effective ML models.  • High computational costs associated with these advanced technologies.  • Underexplored aspects such as the choice of input modality (e.g., image versus mask) and its impact on classification performance.  Addressing these challenges is crucial to enhancing diagnostic accuracy and ultimately improving patient outcomes. (page 3, last paragraph).  **Contribution**  To overcome these limitations, this research proposes a novel Dual-Model Classification Approach (DMCA) that leverages the complementary strengths of convolutional neural networks (CNNs) and deep neural networks (DNNs). The key contributions of this study include:  • Systematic Evaluation: Conducting a comprehensive literature review and evaluation of existing ML techniques for lung cancer classification using two distinct datasets (n=20,000 and n=5,000) to benchmark performance.  • Dual-Model Framework: Developing and implementing the DMCA, which integrates CNNs for image classification with DNNs for mask classification to enhance overall accuracy and efficiency.  • Impact Analysis: Assessing the effects of dataset size and input modality on model performance, with a particular focus on key metrics such as accuracy, sensitivity, and specificity.  • Advancement in Multimodal Classification: Demonstrating that the proposed DMCA achieves superior performance compared to existing approaches, thereby offering a novel methodology that addresses critical gaps in current lung cancer detection and diagnosis strategies.  This study aims to contribute significantly to the field by providing an optimized ML framework that improves lung cancer classification, paving the way for more reliable early detection and better patient outcomes. (Page 5) |
| 4 | The term " support vector machine " is wrongly presented in page no. 7, section 2.2. | The presentation of Support Vector Machine is corrected as below and included within context. |
| 5 | The literature review part is too long. The authors need to concise section 2. | The Literature Review section has been rewritten in concise style, highlighting the grouping of studies by ML models with analytical approach to justify the proposed model in this paper as below:  The Literature Review has been revised as indicated in Point #3 of Reviewer 1's comments |
| 6 | Frame the section 3 as "Research Methodology" | Section 3 has been framed as "3. Research Methodology - The “DMCA” Framework " |
| 7 | SVM model is primarily used for binary classification. In figure 1, the authors included 6 classes (multi-class) classification. Need a clarification. | The feature maps were flattened into a single linear vector before classification. The clarification is described below  The CT scans from The LIDC dataset were stored in the DICOM format for each case that was accompanied by an XML file. Four expert thoracic radiologists who took part in the two-phase imagine annotation procedure provide their insights in the XML file. It includes detailed information regarding the patient, nodule annotations such as nodule location (x, y, z coordinates), nodule diameters (maximum, minimum, and mean), nodule characteristics (e.g., lobulation, calcification, and speculation), level of malignancy of lung nodules, and any additional comments the radiologists have made about each patient's condition. Therefore, the XML file is an important resource for understanding the specific meaning of the identified lung nodules. Radiologists use a five-category system to assess the malignancy levels of lung nodules. These categories were: (1) highly unlikely to be cancerous, (2) moderately unlikely to be cancerous, (3) uncertain likelihood to be cancerous, (4) moderately suspected of being cancerous, and (5) highly suspected of being cancerous and (0) considered as benign. Typically, the first two groups are categorized as benign, whereas the last three are considered potentially malignant.  The SVM was employed as a classifier to determine the nodule cancerous classes (1-5). Because the SVM cannot handle a 2-dimensional approach, we transformed it using the flattening stage to achieve a single vector linear output to implement the SVM model. |
| 8 | A detailed discussion on data augmentation shoulde be presented. | **Data augmentation** techniques are typically applied when the original dataset is insufficient, the study lacks a controlled environment, computational constraints arise, there is a potential risk of overfitting, the data's precision is weak, or the robustness of the models is uncertain.  However, in this research, data augmentation techniques were deliberately not utilized, ensuring that the experiments were conducted both efficiently and effectively |
| 9 | Without transfer learning approach, how authors achieved such performance? Need a justification. | In our study, transfer learning is unsuitable for this research due to the absence of pretrained models tailored to lung CT scans, limiting feature extraction relevance. The unique dual-model approach, integrating image and mask data, requires domain-specific learning for optimal performance. Additionally, pretrained models may not generalize well across different modalities, leading to suboptimal accuracy. The research benefits from training from scratch, allowing better adaptation to dataset characteristics and mitigating biases. Furthermore, transfer learning’s reliance on generic features may overlook subtle lung cancer patterns. Given the study’s focus on high accuracy and scalability, DMCA model ensures improved performance and reliability.  Below are more details  1. **Robust Original Dataset:** Our dataset was comprehensive and diverse, providing sufficient data for effective model training without the need for additional pre-trained models.  2. **Controlled Environment:** The study was conducted in a highly controlled environment, which ensured that the data accurately represented the necessary variations for training.  3. **Model Optimization:** We optimized our models through hyperparameter tuning, regularization, early stopping, and cross-validation to enhance performance  4. **Dataset Size:** there is enough data for training a robust model from scratch, transfer learning can leverage knowledge from a model trained on a larger dataset.  5. **Data Preprocessing:** This included normalization, resizing, noise reduction, and segmentation of CT images. These steps ensured high-quality data and efficient model training, contributing to significant performance and improved accuracy.  6. **Computational Resources:** We utilized advanced computational resources that enabled extensive training and validation processes, ensuring the robustness of our models.  7. **Algorithm Selection:** The choice of algorithms (CNN, DNN, and SVM) and their configurations were tailored to maximize performance based on the characteristics of our dataset.  By leveraging these strategies, we achieved significant performance metrics without the need for transfer learning. |
| 10 | There is a huge confusion in experiment and results section. The authors should include key tables in the result section. The remaining part should be included in supplementary part. | The experiment and results sections have been re-written to address the points raised. Key tables have been included in the results section. Table 2 - Metrics values (Accuracy, F1 Score, Recall, and Precession) - Experiment 1 (Page 33) \| \| **20k Sample Images/Masks - Per Modality** \| \| \| \| \| \|; \| \| **DMCA** \| **CNN - Image** \| **CNN - Mask** \| **DNN - Image** \| **DNN - Mask** \| **SVM - Image** \| **SVM - Mask**; **Accuracy** \| \| 91.21% \| 90.73% \| 63.41% \| 68.09% \| 87.50% \| 84.88% \| 73.30%; **F1 Score** \| \| 91.18% \| 90.81% \| 46.78% \| 66.34% \| 87.30% \| 84.78% \| 72.79%; **Recall** \| \| 91.83% \| 91.35% \| 63.84% \| 68.55% \| 88.09% \| 85.46% \| 73.80%; **Precession** \| \| 90.53% \| 90.28% \| 36.91% \| 65.75% \| 86.53% \| 84.11% \| 71.80% Table 3 - Metrics values (Accuracy, F1 Score, Recall, and Precession) - Experiment 2 (Page 33) \| \| **5k Sample Images/Masks - Per Modality** \| \| \| \| \| \|; \| \| **DMCA** \| **CNN - Image** \| **CNN - Mask** \| **DNN - Image** \| **DNN - Mask** \| **SVM - Image** \| **SVM - Mask**; **Accuracy** \| \| 98.04% \| 97.03% \| 81.13% \| 81.97% \| 91.87% \| 93.29% \| 84.95%; **F1 Score** \| \| 98.01% \| 97.04% \| 65.40% \| 80.22% \| 91.68% \| 93.19% \| 84.44%; **Recall** \| \| 98.66% \| 97.80% \| 81.56% \| 80.81% \| 92.47% \| 93.87% \| 85.46%; **Precession** \| \| 97.37% \| 96.30% \| 54.63% \| 79.63% \| 90.90% \| 92.52% \| 83.46% |
| 11 | The authors need to include statistical analysis for uncertainty evaluation. | To include statistical analysis for uncertainty evaluation, we used ANOVA to determine significant differences between groups and quantify variability within and between groups. This provided a comprehensive understanding of the model's performance and quantified the uncertainty in our results. |
| 12 | Section 7 should present limitations. | The Limitations are: -  **Section 7, "In Conclusion and Future Work"**  limitations were encountered, including computational demands due to large sample sizes, limited information from segmentation masks, and potential biases in the dataset. These challenges restrict the model's generalization. Future work should focus on improving image quality, expanding datasets, and addressing segmentation limitations to further advance lung cancer detection. (Page 36, 2nd Paragraph) |

All Models / All Metrics (Exper. 1, "20k")

|  | Precesion | Recall | F1 Score | Accuracy |
| --- | --- | --- | --- | --- |
| DMCA | 90.542% | 91.8335% | 91.184% | 91.214% |
| CNN Images | 89.992% | 91.4992% | 90.736% | 90.73% |
| CNN Masks | 36.916% | 63.8407% | 46.776% | 63.41% |
| DNN Images | 65.75% | 66.9329% | 66.3351% | 68.0872% |
| DNN Masks | 86.524% | 88.0903% | 87.3016% | 87.496% |
| SVM Images | 84.106% | 85.4605% | 84.778% | 84.884% |
| SVM Masks | 71.804% | 73.804% | 72.786% | 73.296% |

All Models / All Metrics (Exper. 2, "5k")

|  | Precesion | Recall | F1 Score | Accuracy |
| --- | --- | --- | --- | --- |
| DMCA | 97.3704% | 98.6619% | 98.0119% | 98.0424% |
| CNN Images | 96.2967% | 96.8039% | 96.5475% | 97.0347% |
| CNN Masks | 54.634% | 81.5587% | 65.4038% | 81.128% |
| DNN images | 79.6288% | 80.8117% | 80.2158% | 81.966% |
| DNN Masks | 90.8988% | 92.4651% | 91.6752% | 91.8708% |
| SVM Images | 92.5167% | 93.8712% | 93.189% | 93.2947% |
| SVM Masks | 83.458% | 85.458% | 84.4384% | 84.95% |
