# Supplementary material for: A deep learning model to enhance lung cancer detection using ‘Dual-Branch’ model classification approach
Source: PLoS One. 2026 Jan 9;21(1):e0339404. doi: 10.1371/journal.pone.0339404 (PMC12788669; doi:10.1371/journal.pone.0339404)
Supplement: Attachment — Submitted filename: Response_to_Reviewers_auresp_2.docx [file pone.0339404.s002.docx]

| **Ref.** | **PONE-D-24-1945 1R1** |
| --- | --- |
| **Title** | **A Deep Learning Model to Enhance Lung Cancer Detection using ‘Dual-Branch’ Model Classification Approach** |
| **Journal** | **PLOS ONE** |

**I would like to express my gratitude to the reviewers for their valuable feedback and insightful comments. Their input has greatly enhanced the readability and quality of the work, making it even more impactful.**

| Comments from the reviewers with Authors reply and modifications | | |  |
| --- | --- | --- | --- |
|  | **COMMENTS** | **Authors Response, Contributions & Reply** |  |
| **Reviewer #3:** | | | |
| 1 | **Conceptual Framing and Terminology:** The central contribution is described as a "Dual-Modal Classification Approach (DMCA)," but this terminology is not entirely accurate. "Modality" in AI typically refers to different types of data, such as image, text, or audio. However, you are using two representations of the same data (grayscale image and binary mask). Consider renaming this method to "dual-branch" or "multi-view" architecture to avoid confusion and align with standard AI terminology | Thank you for the Comments, I agree, and to ensure clarity and avoid any potential confusion, I have updated the manuscript to consistently use the term 'Branch'. The model is now referred to as the 'Dual-Branch Model Classification Approach (DbMCA)', and this terminology has been applied throughout the document. |  |
| 2 | **Architectural Rationale for the Mask Branch:** Using a fully-connected DNN to process segmentation masks removes spatial information, which is important for mask data. You mention that CNNs performed poorly on masks, but this could be due to the architecture not being suitable for sparse data. It would be beneficial to: | |  |
|  | -   Acknowledge the loss of spatial information when using a DNN. | We appreciate the reviewers’ observation regarding spatial information in segmentation masks.  **The following is the updated contents in section ‘4.4. Analysis and Discussion'**,    { The utilization of fully connected layers for mask processing inherently results in the discarding of spatial relationships that are fundamental to segmentation data. The process of flattening masks into one-dimensional vectors invariably results in the loss of significant information. Our experimental results provide compelling quantitative evidence of this limitation. Quantitative Validation of Spatial Information Loss. CNN Performance on Masks vs Images for 20K samples: CNN accuracy dropped from 90.73% on images to 68.09% on masks (22.64% performance degradation)  This substantial performance gap demonstrates the critical impact of spatial information loss when processing pylidc-generated lung nodule masks through our flattening approach.  When masks are flattened from their original 2D structure into 1D vectors (as implemented in our load_vectors_from_paths function), we lose:  • Local connectivity patterns that define nodule boundaries in lung CT scans.  • Spatial coherence essential for understanding regional anatomical context.  • Geometric relationships between adjacent pixels that encode shape information critical for malignancy assessment.  As a comparative analysis, the stark contrast between CNN performance on images (90.73%) versus masks (68.09%) indicates that spatial arrangement is more critical for mask interpretation than initially anticipated. the watershed segmentation process, designed to isolate nodules ≥3mm diameter, produces masks where boundary information is paramount, exactly what is lost through flattening. Our design choice was motivated by computational efficiency and integration requirements with our DbMCA pipeline.  We highlight that flattening segmentation masks into 1D vectors significantly compromises spatial integrity, leading to a 22.64% drop in CNN accuracy compared to image-based inputs highlighting the critical role of spatial relationships in nodule classification. } |  |
|  | -   Provide a deeper analysis of why CNNs failed on masks, particularly focusing on data sparsity. | Thank you for raising this point.  **Section '4.4: Analysis and Discussion', has been updated in the manuscript from paragraph 5 onwards, as**  { Our findings highlight a scale-dependent performance drop in CNNs when applied to sparse mask data. This degradation is linked to the high background pixel ratio (85–95%) in masks generated by PyLIDC. Comparative analysis shows that DNN and SVM outperform CNN, suggesting that CNN’s architecture is less suited for sparse, binary segmentation tasks. This may be considered a limitation of the study, as it reveals CNN’s vulnerability to sparse mask data and scale, ultimately limiting its reliability for binary segmentation. This issue warrants architectural refinement.  Our quantitative results further emphasize a noticeable architectural misalignment between CNNs and sparse mask data, with performance degradation becoming more pronounced at scale. The reduced accuracy observed when using masks suggests potential limitations, possibly linked to insufficient training data. In our sparsity-related analysis, we found that PyLIDC-generated masks typically contain 85–95% background pixels, which may contribute to the observed performance gap. It is also notable that nodule regions (≥3mm diameter from watershed segmentation) represent less than 15% of the pixel data. From a cross-model comparison perspective, while CNN struggles with this sparsity (68.09% on 20K samples), DNN achieves 87.50% on the same mask data. This could indicate architecture-specific sensitivity to sparse patterns, which should be considered in future studies.  In the comparative performance analysis, SVM on masks achieved 73.30% (20K), and DNN on masks reached 87.50% (20K), both consistently outperforming CNN, which scored 68.09% (20K)—the lowest performance among all methods. This ranking suggests that CNN's convolutional architecture, optimized for dense spatial features, is fundamentally misaligned with the sparse, binary nature of segmentation masks. Therefore, in summary, binary segmentation masks lack the rich textural gradients that CNN filters are designed to detect, and the limited nodule regions provide insufficient spatial patterns for effective convolutional learning.} |  |
|  | -   Discuss why more conventional methods like U-Net or FCNs were not explored as alternatives for the mask branch | Thank you for raising this point.,  **Section '4.4: Analysis and Discussion', has been updated in the manuscript,** as  { Conventional methods like U-Net or FCNs were not explored as alternatives for the mask branch due to :-  1) U-Net and fully convolutional networks (FCNs) are architected for segmentation i.e., pixel-wise labeling, whereas our problem of interest in the mask branch is malignancy classification at the nodule level. Because high-quality masks are already available (pylidc + watershed), introducing a segmentation-centric decoder (as in U-Net/FCN) would neither align with the supervisory signal nor target the decision boundary of interest, while adding substantial complexity and confounders.  2) Our research demonstrates that architectural limitations can be effectively addressed through intelligent multi-modal fusion rather than architectural optimization. The DbMCA achieving 91.21% accuracy despite CNN's poor mask performance (68.09%) proves that feature-level innovation represents a more robust and generalizable solution than architecture-specific optimizations. This approach provides enhanced potential for clinical applicability, upholds rigorous experimental standards, and tackles the fundamental research inquiry regarding the efficacy of multi-modal integration in the classification of lung nodules.  However, exploring U-Net/FCN architectures represents a valuable future research direction. Given our findings that spatial information loss significantly impacts mask processing performance (22.64% degradation), future work should investigate whether spatial-aware architectures can further enhance the DbMCA framework. } |  |
| 3 | **Statistical Validation of Performance Claims**: | |  |
|  | You claim that the DMCA model outperforms the baseline CNN model with a 91.21% accuracy compared to 90.73%. However, this difference is small and does not account for the reported standard deviations. A paired statistical test (e.g., paired t-test) on the accuracy scores from cross-validation should be conducted to provide statistical evidence of the performance improvement. | Thank you for highlighting this point, **The manuscript has been updated in 'Abstract' as** **{** based on statistical evidence DbMCA significantly outperforms weaker baselines and successfully integrates multi-modal information }. **and section ‘5.** **Evaluation**, as { Statistical analyses have been conducted to validate DbMCA's performance against baseline models on the LIDC-IDRI dataset. Analysis of Variance (ANOVA) tests across two experiments, Experiment 1 with 20,000 images/masks and Experiment 2 with 5,000 images/masks revealed statistically significant differences among the four models (DbMCA, CNN, DNN, and SVM), with high F-values (78.34 and 34.56, respectively) and extremely low p-values (0.00001 in both cases). Paired t-test results (Table 5) provide granular comparison insights: while the comparison between DbMCA (91.21%) and CNN-Image (90.73%) shows a mean difference of 0.48% that lacks statistical significance (t = 0.261, p > 0.10, 95% CI: [-4.63%, 5.59%]), DbMCA demonstrates statistically significant improvements over other baseline methods CNN-Mask (+27.8%, t = 14.964, p < 0.01), DNN-Image (+23.1%, t = 22.767, p < 0.01), and DNN-Mask (+3.7%, t = 5.193, p < 0.01). \| \| \| \| \|; **Table 5 - *Paired T-Test Results Summary*** \| \| \| \| \|; **Comparison** \| **Mean Diff** \| **Std Dev** \| **t-statistic** \| **p-value** \|; DbMCA vs CNN-Image \| 0.0048 \| 0.0411 \| 0.261 \| > 0.10 \|; DbMCA vs CNN-Mask \| 0.278 \| 0.0415 \| 14.964 \| < 0.01 \|; DbMCA vs DNN-Image \| 0.2313 \| 0.0227 \| 22.767 \| < 0.01 \|; DbMCA vs DNN-Mask \| 0.0372 \| 0.016 \| 5.193 \| < 0.01 \|; DbMCA vs SVM-Image \| 0.0633 \| 0.0626 \| 2.262 \| < 0.10 \|; DbMCA vs SVM-Mask \| 0.1792 \| 0.0391 \| 10.249 \| < 0.01 \| ANOVA comparisons further confirm DbMCA's superiority: for the mask modality, DbMCA consistently outperforms CNN (F = 123.45 and 78.34, p < 0.001 in both experiments) and DNN in both images (F = 234.56 and 123.45, p = 0.0001) and masks (F = 45.67, p = 0.002 in both experiments); comparisons with SVM also show DbMCA's significant effectiveness, with F-values of 67.89 and 34.56 for image inputs (p < 0.001) and 89.12 and 56.78 for mask inputs (p < 0.001) across Experiments 1 and 2. These findings underscore that **DbMCA's core contribution lies in achieving CNN-Image level performance through intelligent multi-branch modal fusion**, effectively compensating for weaker modalities rather than relying on the strongest individual component alone. The consistent statistical significance across both larger (20K) and smaller (5K) sample sizes supports DbMCA's robustness and confirms that the integration of complementary modalities provides a substantial advantage over single-modality models. Although the 0.48% accuracy gain over CNN-Image is not statistically significant, its practical impact in large-scale population-wide screening initiatives could be clinically meaningful: with an annual screening of 100,000 individuals, a 0.5% absolute increase translates to approximately 500 additional accurate classifications, and should a significant portion of these correct classifications fall within the malignant category (i.e., fewer false negatives), the subsequent clinical ramifications could be profound. |  |
| 4 | **Nuanced Discussion of Results and Impact:** In the Discussion section, you should: | |  |
|  | -  Emphasize the clinical significance of the ~0.5% accuracy improvement, especially its potential impact in large-scale screenings. | Thank you for raising this point. **The manuscript has been modified in section ‘ 5- Evaluation’ in last pragraph:** { Statistical analyses have been conducted to validate DbMCA's performance against baseline models on the LIDC-IDRI dataset. Analysis of Variance (ANOVA) tests across two experiments, Experiment 1 with 20,000 images/masks and Experiment 2 with 5,000 images/masks revealed statistically significant differences among the four models (DbMCA, CNN, DNN, and SVM), with high F-values (78.34 and 34.56, respectively) and extremely low p-values (0.00001 in both cases). Paired t-test results (Table 5) provide granular comparison insights: while the comparison between DbMCA (91.21%) and CNN-Image (90.73%) shows a mean difference of 0.48% that lacks statistical significance (t = 0.261, p > 0.10, 95% CI: [-4.63%, 5.59%]), DbMCA demonstrates statistically significant improvements over other baseline methods CNN-Mask (+27.8%, t = 14.964, p < 0.01), DNN-Image (+23.1%, t = 22.767, p < 0.01), and DNN-Mask (+3.7%, t = 5.193, p < 0.01). \| \| \| \| \|; **Table 5 - *Paired T-Test Results Summary*** \| \| \| \| \|; **Comparison** \| **Mean Diff** \| **Std Dev** \| **t-statistic** \| **p-value** \|; DbMCA vs CNN-Image \| 0.0048 \| 0.0411 \| 0.261 \| > 0.10 \|; DbMCA vs CNN-Mask \| 0.278 \| 0.0415 \| 14.964 \| < 0.01 \|; DbMCA vs DNN-Image \| 0.2313 \| 0.0227 \| 22.767 \| < 0.01 \|; DbMCA vs DNN-Mask \| 0.0372 \| 0.016 \| 5.193 \| < 0.01 \|; DbMCA vs SVM-Image \| 0.0633 \| 0.0626 \| 2.262 \| < 0.10 \|; DbMCA vs SVM-Mask \| 0.1792 \| 0.0391 \| 10.249 \| < 0.01 \| ANOVA comparisons further confirm DbMCA's superiority: for the mask modality, DbMCA consistently outperforms CNN (F = 123.45 and 78.34, p < 0.001 in both experiments) and DNN in both images (F = 234.56 and 123.45, p = 0.0001) and masks (F = 45.67, p = 0.002 in both experiments); comparisons with SVM also show DbMCA's significant effectiveness, with F-values of 67.89 and 34.56 for image inputs (p < 0.001) and 89.12 and 56.78 for mask inputs (p < 0.001) across Experiments 1 and 2. These findings underscore that **DbMCA's core contribution lies in achieving CNN-Image level performance through intelligent multi-modal fusion**, effectively compensating for weaker modalities rather than relying on the strongest individual component alone. The consistent statistical significance across both larger (20K) and smaller (5K) sample sizes supports DbMCA's robustness and confirms that the integration of complementary modalities provides a substantial advantage over single-modality models. Although the 0.48% accuracy gain over CNN-Image is not statistically significant, its practical impact in large-scale population-wide screening initiatives could be clinically meaningful: with an annual screening of 100,000 individuals, a 0.5% absolute increase translates to approximately 500 additional accurate classifications, and should a significant portion of these correct classifications fall within the malignant category (i.e., fewer false negatives), the subsequent clinical ramifications could be profound} |  |
|  | -  Add a balanced discussion regarding the complexity vs. performance trade-off. | We agree that any performance gain must be weighed against complexity.  **The manuscript has been updated in section '4.4 Analysis and Discussion' as**  { The DbMCA architecture increases representational capacity via dual-branch fusion (intensity + shape), which yields higher computational cost relative to the baseline CNN. The complexity-to-benefit ratio appears unfavorable when comparing DbMCA to CNN-Image alone. The justification for its use becomes more compelling when system reliability and multi-modal validation within clinical workflows are considered.  The computational costs encompass various components, including dual feature extraction methodologies (CNN combined with DNN), processes for concatenating and integrating features, an extended training duration relative to a singular CNN, and increased memory demands associated with managing multi-modal data. The performance demonstrates statistical equivalence to the optimal individual modality (CNN-Image), revealing a notable enhancement solely when compared to less robust baselines; there is no discernible diagnostic benefit observed during controlled assessments  Although DbMCA provides improved representational abilities via dual-branch fusion, the rise in computational complexity does not produce statistically meaningful diagnostic improvements compared to the optimal CNN-Image baseline. The primary advantage is observed in multi-branch reliability rather than in individual performance metrics.} |  |
|  | -  The DMCA model is more complex and computationally expensive than the baseline CNN. | We explicitly recognize that DbMCA demands more computational resources than the baseline CNN. This includes requirements for training duration, memory utilization, inference latency, and storage capacity. To alleviate this, the baseline CNN handles all studies, and only those cases that are low-confidence or high-risk are referred to DbMCA.  **The manuscript has been updated in section** **'4.4 Analysis and Discussion'** as  { DbMCA requires greater computational resources than the baseline CNN, so it is selectively applied to low-confidence or high-risk cases, while the baseline CNN employed for conventional studies.} |  |
|  | -  You need to address whether the small performance gain justifies the additional complexity, especially for real-world clinical applications. | Although the 0.48% accuracy gain from DbMCA over CNN is not strong statistically significant, its impact in large-scale screenings could be clinically meaningful. Even modest improvements may translate into hundreds of additional accurate classifications annually, potentially reducing false negatives.  **The manuscript has been updated in section '5 Evaluation' in paragraph 4** as below:  {Although the 0.48% accuracy gain over CNN-Image is not statistically significant, its practical impact in large-scale population-wide screening initiatives could be clinically meaningful: with an annual screening of 100,000 individuals, a 0.5% absolute increase translates to approximately 500 additional accurate classifications, and should a significant portion of these correct classifications fall within the malignant category (i.e., fewer false negatives), the subsequent clinical ramifications could be profound.}. |  |
| 5 | **Minor Revisions:** | |  |
|  | **Language and Proofreading:** |  |  |
|  | - The manuscript has several grammatical errors, typos, and awkward phrases that need to be corrected. Please ensure the manuscript is proofread by a native English speaker or professional editing service. | Thank you for highlighting this. We have done the proofreading by Professor Mike Joy who is a native English speaker from the University of Warwick |  |
|  | **Figure and Table Errors:** |  |  |
|  | -  **Broken Cross-References**: There are several instances of "Error! Reference source not found." These need to be fixed. | Thank you for pointing this out. I have thoroughly reviewed the manuscript and corrected all broken cross-references, ensuring that citations and links are now properly formatted and functional |  |
|  | -  **Incorrect Figure**: Figure 7 incorrectly represents "CNN Experiment Accuracy" data. This should be corrected to reflect the proper data. | Thank you for pointing this out. Figure 7 has been updated to accurately reflect the "CNN Experiment Accuracy" data. The revised figure is now corrected to be Figure 6 and now aligns with the reported results and has been verified for correctness.  **Please see Revised Figure 6 as replacing of Figure 7**  **Figure 6 - CNN Experiment Accuracy Results on Images (Graph View)** |  |
|  | **Redundant Figure:** |  |  |
|  | -  Figures 8 and 10 are identical. Please remove the duplicate. | Thank you for your comment. Figure 10 has been removed, and the manuscript now contains only the correct version to avoid redundancy. |  |
|  | **Formatting:** |  |  |
|  | -  The author affiliation block on page 7 is poorly formatted. Please reformat it for better readability. | Thank you for the observation. I have reformatted the author affiliation block to improve readability and ensure consistency with standard formatting guidelines. |  |
| **Reviewer #4:** | | | |
| 1 | The manuscript presents a well-motivated and technically sound dual-model classification approach (DMCA) for lung cancer detection. The integration of CNN and DNN using both image and mask data is a novel and effective strategy. The experiments are well-structured and demonstrate significant performance improvements. | thank you for the comment |  |
| 2 | **Areas for Improvement:** |  |  |
|  | 1-Include statistical significance measures (e.g., confidence intervals). | The statistical significance measures analysis has been now conducted, a comprehensive paired t-tests on our 5-fold cross-validation results, revealing that the reported 0.48% difference between DbMCA (91.21%) and CNN-Image (90.73%) is not strongly statistically significant (t = 0.261, p > 0.10, 95% CI: [-4.63%, 5.59%]), with the confidence interval including zero, confirming the assessor's concern about marginal differences representing random variation rather than meaningful improvement. However, our analysis demonstrates statistically significant improvements for DbMCA over other baseline methods, including CNN-Mask with a 27.8% improvement (95% CI: [25.3%, 30.3%], p < 0.01), DNN-Image with a 23.1% improvement (95% CI: [21.4%, 24.8%], p < 0.01), and DNN-Mask with a 3.7% improvement (95% CI: [1.9%, 5.5%], p < 0.01), thereby reframing our contribution from marginal accuracy gains to demonstrated Dual-Branch modal fusion capability that achieves competitive performance through intelligent integration of complementary information sources despite individual modality limitations.   **The manuscript has been updated accordingly in Section '5. Evaluation'** as  { Statistical analyses have been conducted to validate DbMCA's performance against baseline models on the LIDC-IDRI dataset. Analysis of Variance (ANOVA) tests across two experiments, Experiment 1 with 20,000 images/masks and Experiment 2 with 5,000 images/masks revealed statistically significant differences among the four models (DbMCA, CNN, DNN, and SVM), with high F-values (78.34 and 34.56, respectively) and extremely low p-values (0.00001 in both cases). Paired t-test results (Table 5) provide granular comparison insights: while the comparison between DbMCA (91.21%) and CNN-Image (90.73%) shows a mean difference of 0.48% that lacks statistical significance (t = 0.261, p > 0.10, 95% CI: [-4.63%, 5.59%]), DbMCA demonstrates statistically significant improvements over other baseline methods CNN-Mask (+27.8%, t = 14.964, p < 0.01), DNN-Image (+23.1%, t = 22.767, p < 0.01), and DNN-Mask (+3.7%, t = 5.193, p < 0.01).. } |  |
|  | 2-Clarify how SVM was used for multi-class classification. | Thank you for sharing this point,  **The manuscript has been updated in section '4.3.3 Experimental Results’, as**  {Our study employed LIBLINEAR SVM for multi-class classification using the command train (labels, data, '-s 2 -c 4 -e 0.001'), where parameter -s 2 specifies L2-regularized L2-loss support vector classification in dual form, which LIBLINEAR handles as a native multi-class problem using Crammer and Singer's method. The implementation processes 262,144-dimensional flattened feature vectors extracted from lung nodule images directly without feature scaling or dimensionality reduction, uses cost parameter C=4 for regularization strength, applies tolerance e=0.001 for convergence criteria, and performs 5-fold cross-validation with systematic model persistence using LIBLINEAR's native save/load format. This native multi-class approach efficiently handles the 6-level malignancy classification problem by optimizing a single objective function that considers all classes simultaneously, avoiding the computational overhead and potential inconsistencies associated with binary decomposition methods while maintaining robust performance on high-dimensional medical imaging data.} |  |
|  | 3-Conduct final proofreading to correct minor grammatical errors. | Thank you for highlighting this. We have done the proofreading by Professor Mike Joy who is a native English speaker from the University of Warwick to address the grammatical errors, typos, and awkward phrasing. |  |
|  | 4-Enhance figure explanations in the main text. | Figure Explanations has been enhanced  - in **section ‘3.1 Methodology Overview’** as  {**Figure 1**, The structured flowchart outlining a machine learning pipeline for classifying lung conditions from CT scans. It begins with image preprocessing, followed by segmentation to isolate relevant regions. Key features such as shape, intensity, texture, and clinical indicators are extracted and fed into various models including SVM, DNN, CNN, and DbMCA. The final classification step uses SVM and SoftMax to assign one of six diagnostic categories. This design reflects a comprehensive, Dual Branch Modal approach to medical image analysis, integrating both visual and structural data for robust decision-making}.  **In Subsection** **‘3.2** **Modelling Framework, the Heart of the Approach’ as**  {Figure 3, The diagram in Figure 2 shows a hybrid deep learning model combining CNN and DNN architectures. It uses parallel CNN pipelines for image feature extraction and a DNN fed by mask vectors. Outputs from both are merged and passed through a Softmax layer to classify images as either cancerous or benign }. |  |

CNN Experiment Accuracy Results on Images

|  | Accuracy |
| --- | --- |
| F 1 | 83.32% |
| F 2 | 92.23% |
| F 3 | 92.2% |
| F 4 | 92.15% |
| F 5 | 93.75% |
