# Supplementary material for: A deep learning model to enhance lung cancer detection using ‘Dual-Branch’ model classification approach
Source: PLoS One. 2026 Jan 9;21(1):e0339404. doi: 10.1371/journal.pone.0339404 (PMC12788669; doi:10.1371/journal.pone.0339404)
Supplement: Attachment — Submitted filename: Response_to_Reviewers_auresp_3.docx [file pone.0339404.s003.docx]

| **Ref.** | **PONE-D-24-19451R2** |
| --- | --- |
| **Title** | **A Deep Learning Model to Enhance Lung Cancer Detection using ‘Dual-Branch’ Model Classification Approach** |
| **Journal** | **PLOS ONE** |

**I would like to express my gratitude to the reviewers for their valuable feedback and insightful comments. Their input has greatly enhanced the readability and quality of the work, making it even more impactful.**

| Comments from the reviewers with Authors’ replies and modifications | | |
| --- | --- | --- |
|  | **COMMENTS** | **Authors Response, Contributions & Reply** |
| **Additional Editor Comments:** | | |
| 1 | The integration of the DNN and CNN branches is described as occurring through concatenation; however, the fusion weights or strategy applied during this process have not been specified. Clarifying how the two branches are balanced during feature fusion would enhance the methodological transparency. | We sincerely thank the editors for this insightful observation.  **The last paragraph of '3.2 Modelling Framework, the Heart of the Approach' section, page 19, has been updated:** -  [In the current investigation, the integration of the DNN and CNN branches is performed via straightforward feature concatenation, without the application of explicit fusion weights or a learned balancing mechanism. The strategy applied during the process is specified as that the concatenated feature vector is subsequently passed through fully connected layers, which implicitly learn to weigh and integrate the combined features during training. Both branches process the inputs of same type of datasets independently. In this balanced way, methodological transparency is smoothly enhanced, because the expected outputs of the DNN and CNN branches are concatenated along the feature dimension without applying predefined fusion weights. The subsequent fully connected layers are responsible for learning the optimal combination of these features during training.] |
| 2 | The study currently employs an 80/20 train-test data split. Incorporating k-fold cross-validation or using an independent test dataset would help improve the robustness and generalizability of the model’s performance. | We appreciate the editor’s valuable suggestion regarding model validation. **Updated corrections are shown in Sections 4.3 page 29 as per the following:-** [ Addressing the Cross‑Validation and Performance Robustness and to ensure methodological rigor and strengthen both generalizability and robustness, five‑fold cross‑validation was systematically implemented across all models. Each fold employed a distinct, randomly generated partition of the training and test data, ensuring that every sample contributed to both training and evaluation. This procedure was executed using LIBLINEAR’s native persistence format, guaranteeing consistency and reproducibility across folds. By explicitly integrating five‑fold cross‑validation into the experimental design, the study mitigates overfitting and reduces dependence on a single train–test split, thereby enhancing confidence in the reported outcomes. The impact of this validation strategy is reflected in the performance results for the 5K sample dataset. The conducted careful evaluation demonstrate that the Dual‑Branch Model Combined Approach (DbMCA) achieved the highest accuracy (98.04%), F1 score (98.01%), and recall (98.66%), outperforming single‑modality models. CNN‑Image also demonstrated strong performance (accuracy 97.03%, F1 score 97.04%), whereas CNN‑Mask lagged significantly in precision (54.63%) and F1 score (65.40%), suggesting that mask‑only features are less discriminative when isolated. DNN‑based models achieved moderate results (~81–82% accuracy), while SVM‑Image and SVM‑Mask produced competitive outcomes (91.87% and 93.29% accuracy, respectively), confirming the utility of classical classifiers when feature extraction is robust. Taken together, these findings demonstrate that dual-branch model (DbMCA) yields the most generalizable and robust performance, while mask‑only models require refinement to improve precision. The consistent superiority of DbMCA across folds validates the effectiveness of five‑fold cross‑validation in producing stable, unbiased estimates and highlights the model’s potential applicability to broader clinical contexts. ] **Updated corrections are shown in Sections 4.3 page 36 as the following :-** [ The application of five‑fold cross‑validation across all experiments ensured that performance estimates were stable and unbiased, thereby reinforcing both robustness and generalizability. By averaging results over multiple randomized splits, the study minimized the influence of class distribution variability and reduced dependence on any single train–test partition. The impact of this strategy is evident in the comparative results: the Dual‑Branch Model Combined Approach (DbMCA) consistently achieved superior accuracy, F1 score, and recall, outperforming single‑modality models. CNN‑Image performed strongly, while CNN‑Mask exhibited weaker precision, highlighting the limitations of mask‑only features when isolated. Classical classifiers such as SVM produced competitive outcomes yet remained below the performance of DbMCA. Taken together, these findings confirm that multimodal fusion yields the most reliable outcomes, and the consistent superiority of DbMCA across folds underscores its potential applicability in clinical contexts where stability and reproducibility are paramount.] *Table 3 - Metrics values (Accuracy, F1 Score, Recall, and Precession) - Experiment 1* \| **20k Sample Images/Masks - Per Modality** \| \| \| \| \| \| \|; \| **DbMCA** \| **CNN - Image** \| **CNN - Mask** \| **DNN - Image** \| **DNN - Mask** \| **SVM - Image** \| **SVM - Mask** \|; **Accuracy** \| **91.21%** \| **90.73%** \| **63.41%** \| **68.09%** \| **87.50%** \| **84.88%** \| **73.30%** \|; **F1 Score** \| **91.18%** \| **90.81%** \| **46.78%** \| **66.34%** \| **87.30%** \| **84.78%** \| **72.79%** \|; **Recall** \| **91.83%** \| **91.35%** \| **63.84%** \| **68.55%** \| **88.09%** \| **85.46%** \| **73.80%** \|; **Precision** \| **90.53%** \| **90.28%** \| **36.91%** \| **65.75%** \| **86.53%** \| **84.11%** \| **71.80%** \| *Table 4 - Metrics values (Accuracy, F1 Score, Recall, and Precession) – Experiment* \| **5k Sample Images/Masks - Per Modality** \| \| \| \| \| \| \|; \| **DbMCA** \| **CNN - Image** \| **CNN - Mask** \| **DNN - Image** \| **DNN - Mask** \| **SVM - Image** \| **SVM - Mask** \|; **Accuracy** \| **98.04%** \| **97.03%** \| **81.13%** \| **81.97%** \| **91.87%** \| **93.29%** \| **84.95%** \|; **F1 Score** \| **98.01%** \| **97.04%** \| **65.40%** \| **80.22%** \| **91.68%** \| **93.19%** \| **84.44%** \|; **Recall** \| **98.66%** \| **97.80%** \| **81.56%** \| **80.81%** \| **92.47%** \| **93.87%** \| **85.46%** \|; **Precision** \| **97.37%** \| **96.30%** \| **54.63%** \| **79.63%** \| **90.90%** \| **92.52%** \| **83.46%** \| |
| 3 | The authors are encouraged to provide a discussion explaining the observed decrease in accuracy with larger datasets, addressing whether this could be attributed to factors such as class imbalance, model underfitting, or noisy data | We appreciate the editor’s observation; **Last paragraph of section 4.4 , page 42, has been updated with the following:** -  [The marginal decrease in accuracy observed with the larger 20K dataset is not indicative of systematic degradation but rather reflects the increased heterogeneity and complexity introduced by a broader sample of cases. Smaller datasets (5K samples) may inadvertently favor more homogeneous or less challenging cases, yielding higher accuracy. In contrast, expanding to 20K samples exposes the model to a wider range of nodule types, imaging protocols, and radiologist interpretations, thereby increasing variability. While this introduces slight fluctuations in performance, it enhances the model’s ability to generalize to real‑world clinical scenarios. Moreover, the five‑fold cross‑validation strategy ensures that results are averaged across randomized splits, minimizing the impact of class distribution or annotation noise. Regarding *model underfitting,* the architecture was carefully tuned, and early stopping was implemented once the validation loss ceased to improve. This ensured that models were trained to convergence without premature termination. As for *noisy data,* preprocessing steps were explicitly designed to mitigate this challenge. Gabor filters were applied to enhance texture representation, noise reduction and smoothing techniques clarified fine structural details, and grayscale conversion standardized image intensity ranges. These measures ensured consistency across folds and minimized the impact of annotation variability inherent in LIDC‑IDRI.  Thus, the observed variation represents a natural trade‑off between headline accuracy and broader generalizability, underscoring the robustness of the proposed DbMCA model] |
| 4 | Table 5, which presents the ANOVA results, does not specify the sample size used in the statistical analysis. Including this information is essential for clarity and reproducibility. | We thank the editors for highlighting this important detail.  **Updated contents are shown in section ‘5 Evaluation’, page 46 as below:**  Analysis of Variance (ANOVA) tests across two experiments, Experiment 1 with 20,000 images/masks and Experiment 2 with 5,000 images/masks revealed statistically significant differences among the four models (DbMCA, CNN, DNN, and SVM),  Additionally, the ANOVA results for Experiment 2 (5000 Samples) give a more comprehensive comparison across experimental conditions and improve transparency and support the robustness of our statistical evaluation. |
| 5 | A more detailed discussion on the clinical applicability of the proposed DbMCA model in early lung cancer screening workflows is recommended to highlight the potential real-world impact of the study. | We appreciate this valuable suggestion and have expanded the Discussion to highlight the clinical applicability of the proposed DbMCA model.  **The manuscript has been updated in 'Section 4.4 (Analysis and Discussion)', page 43, by adding the following:-**  [The proposed DbMCA model demonstrates clear potential for integration into early lung cancer screening workflows. Its hybrid design, which fuses image‑based features with semantic mask representations, reflects the multifactorial nature of clinical decision‑making. This approach may assist radiologists in reducing diagnostic uncertainty, particularly in borderline or ambiguous cases, and could be incorporated into computer‑aided diagnosis (CADx) systems to enhance triage efficiency.  From a population‑level perspective, the model’s performance gains are clinically significant. In large‑scale screening programs, even modest improvements in accuracy can translate into substantial real‑world benefits. For instance, in a cohort of 100,000 individuals screened annually, a 0.5% absolute increase in accuracy equates to approximately 500 additional correct classifications. If a meaningful proportion of these correspond to malignant nodules, the reduction in false negatives would facilitate earlier detection, improve patient prognosis, and potentially reduce lung cancer mortality.  By integrating both semantic and imaging features, DbMCA model mirrors the complexity of clinical workflows and demonstrates promise as a supportive tool for radiologists in early lung cancer screening] |
